# Chemical Characterization and Bioactivities of Sericin Extracted from Silkworm Cocoons from Two Regions of Portugal

**DOI:** 10.3390/molecules30051179

**Published:** 2025-03-06

**Authors:** Sara Reis, Carina Spencer, Cristina M. Soares, Soraia I. Falcão, Sónia P. Miguel, Maximiano P. Ribeiro, Lillian Barros, Paula Coutinho, Josiana Vaz

**Affiliations:** 1 Research Centre for Active Living and Wellbeing (LiveWell), Instituto Politécnico de Bragança, Campus de Santa Apolónia, 5300-253 Bragança, Portugal; sarareis@ipb.pt; 2 CIMO, LA SusTEC, Instituto Politécnico de Bragança, Campus de Santa Apolónia, 5300-253 Bragança, Portugal; sfalcao@ipb.pt (S.I.F.); lillian@ipb.pt (L.B.); 3 BRIDGES—Biotechnology Research Innovation Design of Health Products, Polytechnic Institute of Guarda, 6300-559 Guarda, Portugal; carinaspencer2017@gmail.com (C.S.); spmiguel@ipg.pt (S.P.M.); mribeiro@ipg.pt (M.P.R.); 4 REQUIMTE/LAQV, Instituto Superior de Engenharia, Instituto Politécnico do Porto, Rua Dr. António Bernardino de Almeida, 431, 4200-072 Porto, Portugal; cds@isep.ipp.pt; 5 CICS-UBI—Health Sciences Research Centre, University of Beira Interior, 6200-506 Covilhã, Portugal

**Keywords:** *Bombyx mori*, silk protein, bioactivity, amino acids, total protein

## Abstract

Sericin has been characterized as demonstrating a variety of bioactivities, establishing it as a valuable resource for biomedical and pharmaceutical applications. The diverse biological activities of sericin are likely linked to its unique biochemical composition and properties. This study aimed to assess the effect of origin, seasonality, and amino acid composition on the bioactivity of sericin samples from two Portuguese regions compared to commercial sericin. The amino acid profile was analyzed using HPLC-FLD. Moreover, several bioactivities were assessed through in vitro assays, including antiproliferative effects, cell migration, antimicrobial activity, anticoagulant properties, antioxidant capacity, and anti-inflammatory effects. The results obtained in this work revealed that the origin and season affect the sericin amino acid profile. In its pure state, sericin exhibited a low content of free amino acids, with tyrosine being the most abundant (53.42–84.99%). In contrast, hydrolyzed sericin displayed a higher amino acid content dominated by serine (54.05–59.48%). Regarding bioactivities, the sericin tested did not demonstrate antioxidant or anti-inflammatory potential in the conducted tests. Notwithstanding, it showed antiproliferative activity in contact with human tumor cell lines at a minimum concentration of 0.52 mg/mL. Regarding antimicrobial activity, sericin had the capacity to inhibit the growth of the bacteria and fungi tested at concentrations between 5 and 10 mg/mL. Additionally, sericin demonstrated its capacity to prolong the coagulation time in pooled human plasma, indicating a potential anticoagulant activity. In addition, the origin and season also revealed their impact on biological activities, and sericin collected in Bragança in 2021 (S3) and 2022 (S4) demonstrated higher antiproliferative, antibacterial, and anticoagulant potentials. Future studies should focus on optimizing sericin’s bioactivities and elucidating its molecular mechanisms for clinical and therapeutic applications.

## 1. Introduction

Some species of worms and other insects, such as spiders and scorpions, can synthesize silk [1]; however, the silk more exploited commercially is obtained from silkworm cocoons [2]. During metamorphosis, *Bombyx mori* produce cocoons for self-protection against adverse environmental conditions and the attacks of biological agents such as birds, insects, and bacteria [3]. The cocoon is constituted by fibroin (silk fibers) that are attached by a protein, sericin, acting as a natural glue [4].

**Figure 1:**
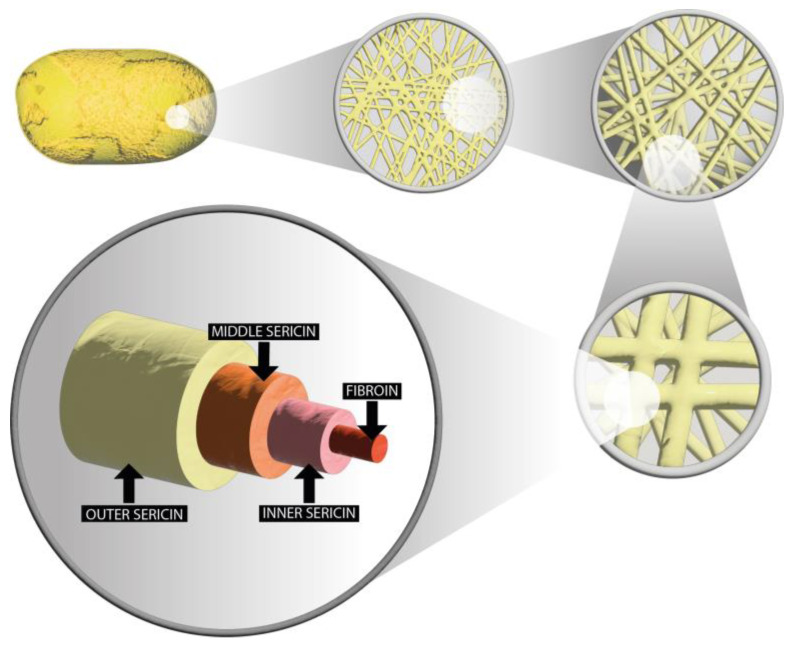
Morphology and structure of the cocoon of *Bombyx mori*, from the complete cocoon to the individual fiber–sericin combination, adapted from [5,7].

Generally, the fibrous silk fibroin makes up 65–85% of the cocoon layer, whereas the sericin layer makes up 15–35%, depending on the variety of silkworm [5]. Thus, fibroin is enveloped by sericin’s inner, middle, and outer layers according to its water solubility (Figure 1). Since the concentration of hydrophilic amino acids gradually rises and the amount of hydrophobic amino acids gradually decreases from the inner to outer sericin layers, hot or boiling water makes the outermost sericin layer the simplest to dissolve [5,6].

Sericin has been studied for a wide spectrum of biological actions due to its biochemical properties, such as antioxidant [8,9,10,11,12,13], anti-inflammatory [8,9,14], antiproliferative [8,15], antimicrobial [9,11,12,13,16,17], hypoglycemic effect [18,19], UV protection [20], anti-hyperpigmentation [20,21], and anti-aging [20]. Additionally, the potential applications of sericin have been studied for different industries, such as biomedical and pharmaceutical, food, textile, and others [3,22,23,24]. Biomedical applications considering the biological activities of sericin have recently been reviewed in a previous article [24].

This work aimed to evaluate the effect of season and origin on sericin’s biochemical profile and biological activities. For this, sericin samples of different origins in Portugal and seasons were compared with sericin standards by characterizing their amino acid profiles and bioactivities, such as antiproliferative and cytotoxic activities, antioxidant activity, anti-inflammatory activity, and antimicrobial activity.

## 2. Results

### 2.1. Chemical Characterization

Silkworm cocoons are mainly composed of fibroin and sericin. Sericin was extracted using an autoclave at 120 °C for 30 min. Sericin samples S1 and S2, from Castelo Branco and Bragança, respectively, were extracted in February 2022, and S3 and S4, from Bragança, were extracted in September 2022. The yields obtained ranged from 19 to 30.2%. This difference in values could potentially be linked to the moisture content of each cocoon. S4, a sample of the cocoons collected in Bragança in 2022, had the highest extraction yield (30.2%). Sample S3 presented the second-highest yield of sericin (26.7%) and was representative of the cocoons collected in Bragança in 2021. Finally, samples S1 and S2 from Castelo Branco and Bragança in 2019 exhibited identical extraction yields (19%), being the lowest yielding. Additionally, two commercial sericin samples, SC1 and SC2, were analyzed and compared with the laboratory-extracted samples in terms of their amino acid profile and bioactivities.

The total and free amino acids were analyzed using high-performance liquid chromatography with a fluorescence detector (HPLC-FLD) following derivatization, with or without hydrolysis. A total of 7 amino acids were identified in the commercial samples in the non-hydrolyzed sericin samples, while the sericin extracts obtained contained between 10 and 13 amino acids. In contrast, 13 to 16 amino acids were identified in the hydrolyzed samples.

The non-hydrolyzed samples (free amino acid content) showed lower percentages of free amino acids than the hydrolyzed samples. The tyrosine (Tyr) levels ranged from 53.42% (S1) to 84.99% (SC1), while in S4, Tyr was below the limit of detection (<LOD). Serine (Ser) was more abundant in the non-hydrolyzed samples, with levels between 8.55% (SC2) and 17.94% (S3), but was also below the detection limit in S4. The glutamic acid (Glu) levels varied significantly, with the highest concentrations in S1 (19.92%) and S2 (17.23%) and the lowest in SC2 (0.85%). Notably, S4 was the only sample containing aspartic acid (Asp) and cysteine (Cys).

Following hydrolysis (total amino acid content), a significant increase in the free amino acid concentrations was observed. The Ser concentrations increased from 54.05% (S1) to 59.48% (SC1). In contrast, the Tyr levels decreased significantly compared to the non-hydrolyzed state, ranging from 6.85% (S3) to 8.13% (S1). In SC1 and SC2, where Tyr was initially the most abundant amino acid, its concentration fell below the detection limit after hydrolysis. Asp was also present at higher levels in the hydrolyzed samples, with concentrations between 9.45% (S2) and 12.00% (SC2), whereas it was mostly absent in the non-hydrolyzed samples, except for S4 (1.42%). Interestingly, Cys, which was previously detected at high levels in S4 (79.16%), was no longer present after hydrolysis.

Table 1 show the amino acid profiles of the samples, highlighting that Ser, Glu, Gly, and Tyr were the most abundant in both hydrolyzed and non-hydrolyzed forms. In non-hydrolyzed forms, sample S4 exhibited higher concentrations of Asp (1.42%) and Cys, while samples S1 and S2 had the highest Glu levels (19.92% and 17.23%, respectively). Asparagine (Asn), alanine (Ala), leucine (Leu), and valine (Val) were present in S1, S2, and S3. Lysine (Lys) was identified in SC2 and S4, while isoleucine (Ile) was found in S3.

### 2.2. Bioactivity Analysis

#### 2.2.1. Antiproliferative Activity

The results obtained for the sulforhodamine B (SRB) assay, conducted on five human tumor cell lines, such as AGS (epithelial cells from stomach tissue with gastric adenocarcinoma), CaCo-2 (epithelial cells isolated from colon tissue derived from a male with colorectal adenocarcinoma), MCF-7 (epithelial cells from breast tissue with ductal carcinoma), NCI-H460 (epithelial cells from pleural fluid with large cell lung cancer), and HeLa (epithelial cells from cervix with adenocarcinoma), demonstrated that sericin samples S1 to S4 inhibited tumor cell proliferation at concentrations ranging from 0.520 to 1.519 mg/mL (Table 2). In contrast, commercially sourced sericin (SC1 and SC2) showed no inhibitory effect on cell growth, even at the maximum concentration tested.

All sericin samples (S1–S4) successfully inhibited the proliferation of the AGS cell line, with sample S4 exhibiting the lowest GI_50_ value (0.791 ± 0.024 mg/mL), indicating the strongest antiproliferative activity. For the Caco-2 cell line, only samples S3 and S4 inhibited cell growth, with S4 showing a significantly lower GI_50_ value (0.814 ± 0.03 mg/mL), suggesting higher efficacy. Regarding the MCF-7 cell line, sample S2 did not exhibit any inhibitory activity, whereas samples S1, S3, and S4 were effective, with S4 presenting the most potent effect (GI_50_ = 0.520 ± 0.032 mg/mL). All four sericin samples inhibited the proliferation of the NCI-H460 cell line, although there were no statistically significant differences between them. Finally, samples S3 and S4 demonstrated antiproliferative activity against the HeLa cell line, but their GI_50_ values were not significantly different (*p* < 0.05).

#### 2.2.2. Antioxidant and Anti-Inflammatory Activities

The sericin samples did not exhibit antioxidant or anti-inflammatory activities. For antioxidant activity, two assays were performed: the thiobarbituric acid reactive substances (TBARS) method, with concentrations ranging from 2.18 mg/mL to 70 mg/mL, and the Cell Antioxidant Activity (CAA) assay, with tested concentrations between 500 and 2000 μM. Anti-inflammatory activity was assessed using the nitric oxide (NO) inhibition assay, with four tested concentrations between 1.6 mg/mL and 0.02 mg/mL. Higher concentrations were not tested, as they are irrelevant for pharmaceutical and biotechnological applications due to their cytotoxicity.

#### 2.2.3. Antimicrobial Activity

The minimal inhibitory concentration (MIC) and minimal bactericidal concentration (MBC) obtained for clinical bacteria, Gram-negative and Gram-positive strains, are shown in Table 3.

For clinically relevant bacterial strains, the samples exhibited superior MIC values against Gram-negative bacteria. The bacterial growth inhibition values for the sericin samples ranged from 5 to 10 mg/mL.

Samples S1 and S2 inhibited the growth of *E. coli* and *Y. enterocolitica* at a concentration of 10 mg/mL. Sample S3 inhibited the growth of *E. coli* at 5 mg/mL and that of *P. aeruginosa*, *S. enterica*, *Y. enterocolitica*, and *L. monocytogenes* at 10 mg/mL. Sample S4 inhibited the growth of all tested strains except *B. cereus*, with notable inhibition of *E. coli* at 5 mg/mL, while the remaining strains were inhibited at 10 mg/mL. Sample SC1 inhibited the growth of *E. coli*, *P. aeruginosa*, and *S. aureus* at 10 mg/mL. Finally, sample SC2 only inhibited the growth of *E. coli* at 10 mg/mL.

#### 2.2.4. Anticoagulant Activity

The table below (Table 4) shows the results obtained for the anticoagulant activities of the different samples of sericin via the activated partial thromboplastin clotting time (APTT) and prothrombin time (PT) methods.

Sericin increased the clotting time for the first two concentrations of the PT test. In contrast to the other samples, S2 and S4 exhibited a longer clotting time. There were no significant differences among the remaining samples or when compared to the control, even though they delayed the clotting time at these concentrations.

On the other hand, all sericin samples significantly extended the clotting time in the APTT assay compared to the control at concentrations of 1.6 and 0.4 mg/mL. Although all the samples prolonged the coagulation time in the subsequent concentrations, samples S4, SC1, and SC2 exhibited significant differences compared to the control.

#### 2.2.5. Cytotoxic Activity

**Figure 2:**
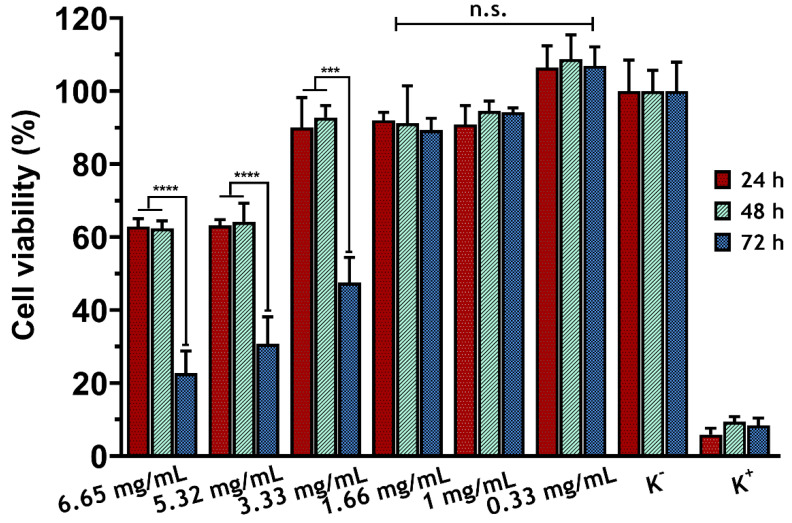
Determination of the cell viability of fibroblasts incubated with different sericin concentrations (0.33, 1, 1.66, 3.33, 5.32, and 6.65 mg/mL) through MTT assay, after 24, 48, and 72 h of incubation. Data are presented as the mean ± standard deviation; n = 5, *** *p* < 0.001; **** *p* < 0.0001; n.s.: non-significant.

The study confirmed the biocompatibility on normal human dermal fibroblasts (NHDF) using the MTT assay for concentrations below 1.66 mg/mL. Furthermore, the MTT test demonstrated the cell viability of human fibroblasts when incubated with sericin for 24, 48, and 72 h (Figure 2).

Notably, the two highest concentrations of sericin tested (6.65 mg/mL and 5.32 mg/mL) induced cytotoxic effects immediately after 24 h of incubation, as the percentage of cell viability fell below 70%. Conversely, the concentration of 3.33 mg/mL only induced this cytotoxic effect after 72 h of incubation.

#### 2.2.6. Scratch Assay

**Figure 3:**
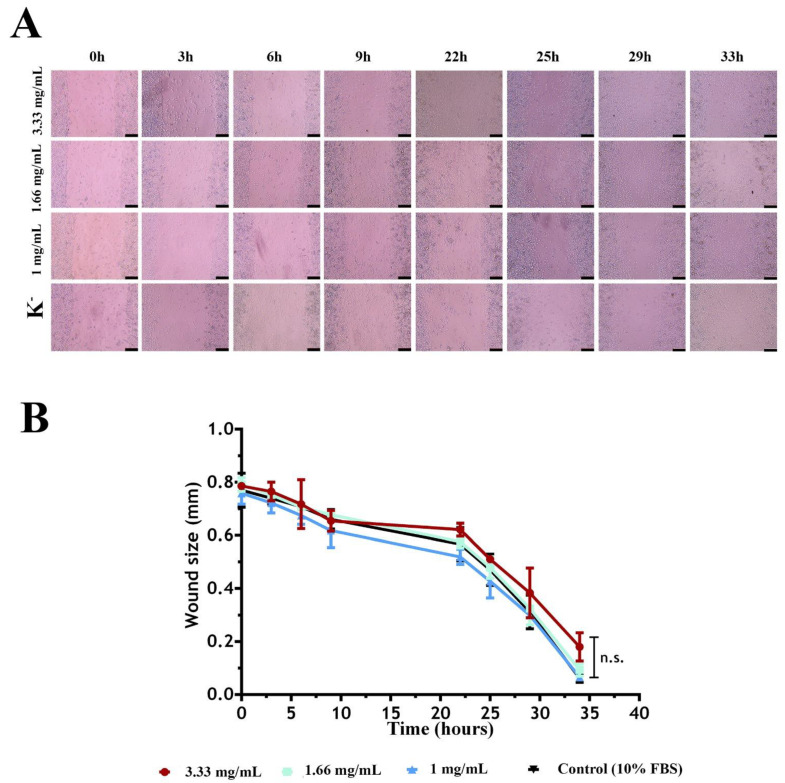
In vitro scratch assay. (**A**) Microscopic images of the scratch test, representing the migration of human fibroblasts induced by different concentrations of sericin (1.66 mg/mL and 1 mg/mL) and by medium supplemented with 10% FBS (negative control) at different incubation times (0 h, 3 h, 6 h, 9 h, 22 h, 25 h, 29 h, and 33 h). (**B**) Effect of sericin on the migratory activities of fibroblasts in the scratch assay by measuring the wound size (mm) at different timepoints. The scale bar corresponds to 200 µm. Data are presented as the mean ± standard deviation, n = 3; n.s.: non-significant.

This assay assessed sericin’s capacity to stimulate fibroblast migration. To achieve this, three concentrations of sericin (3.33, 1.66, and 1.00 mg/mL) were incubated with human fibroblasts, and their effects were compared with those of culture medium supplemented with 10% fetal bovine serum (FBS). These concentrations were selected based on the cytotoxicity results. Considering their biocompatibility with NHDF, these concentrations were chosen for the cell migration assay to evaluate their potential application as regeneration promoters. Over 33 h, different microscopy images were acquired to determine the level of in vitro wound closure over time (Figure 3).

Sericin induced fibroblast migration at all concentrations tested, with no significant differences between the tested concentrations and FBS. However, it is important to note that the lower concentration of sericin tested (1 mg/mL) made it possible to improve wound closure in vitro and compared to the other groups (Figure 3A).

## 3. Discussion

In this study, sericin samples from different regions of Portugal, specifically Castelo Branco and Bragança, collected over various seasons, were extracted using a physical method involving high temperature and pressure. These samples were compared with two commercial sericin products regarding their amino acid profiles and bioactivities, including antiproliferative, cytotoxic, antioxidant, anti-inflammatory, antimicrobial, anticoagulant, and cell migration activities.

Regarding the sericin extraction yield, our results align with previous findings. For instance, Gimenes et al. [25] reported a yield of 21.99 ± 0.96% from *B. mori* cocoons using hot-water extraction at 120 °C. Gimenes and collaborators also achieved a higher yield of 30.04 ± 0.83% using 0.5% Na_2_CO_3_ solution. Similarly, Aramwit et al. [26] obtained yields ranging from 17.00 ± 3.14 to 21.47 ± 0.62 from different strains of *B. mori* cocoons (white, greenish, and yellow shells) using the same method employed in this study.

It is well documented that the extraction method significantly influences the sericin yield [27,28], alongside the origin of the cocoon and the strain of silkworm [3]. In this study, the differences in the extraction yields could be influenced by factors such as location, collection period, and relative humidity, as the extraction method and *Bombyx* species were consistent across all samples. Further studies are required to confirm these findings.

The amino acid analysis, conducted via HPLC-FLD with and without hydrolysis, revealed distinct profiles in the commercial and extracted sericin samples. Non-hydrolyzed commercial sericin samples exhibited fewer amino acids, identifying only seven. In contrast, the sericin extracts derived in this study presented a broader spectrum, ranging from 10 to 13 amino acids. After hydrolysis, the amino acid diversity increased further, with 13 to 16 amino acids being detected, underscoring the efficacy of hydrolysis in freeing bound amino acids from the substrate.

The comparison between non-hydrolyzed and hydrolyzed sericin samples revealed that free amino acids were significantly lower in the non-hydrolyzed state, with variations in Ser, Gly, and Glu levels suggesting differences in the binding or abundance of these amino acids. Hydrolysis led to a marked increase in free amino acids, particularly Ser, while Gly levels decreased. Glu remained more stable across hydrolyzed samples, and Asp was absent in most non-hydrolyzed samples and increased significantly post-hydrolysis. Further highlighted in the amino acid profiles, these variations underscore the influence of cocoon origin and seasonality on sericin composition.

The observed increase in free amino acids post-hydrolysis can be explained by the breakdown of glycosidic and peptide bonds during the hydrolysis process. This aligns with previous studies indicating that hydrolysis facilitates the quantitative liberation of amino acids from sericin substrates [29,30]. As a result, hydrolysis increases the concentration of free amino acids and alters the overall amino acid profile.

Several authors used hot water under standard pressure to analyze the amino acid compositions for sericin samples extracted from *Bombyx mori* cocoons. Namely, Kunz et al. [31] identified 17 amino acids in sericin extracted from Brazilian cocoons, with serine accounting for 30.37%, glycine for 27.16%, and aspartic acid for 14.5%. Keawkorn et al. [32] reported 18 amino acids from Thai cocoons, with Ser at 33.4%, Asp at 16.7%, and Gly at 13.5%. Similarly, Ampawong et al. [33], identified 17 amino acids from Thai cocoons, with serine at 33.63%, aspartic acid at 15.64%, and glycine at 15.03%. These findings corroborate our results, especially concerning the prevalence of Ser as the most abundant amino acid in hydrolyzed sericin.

The differences in amino acid profiles observed in this study, particularly between hydrolyzed and non-hydrolyzed sericin, can be attributed to multiple factors. In addition to the impact of the hydrolysis process on the protein composition, variations in the cocoon origin and harvest year also significantly influenced the amino acid content. Despite the consistency of the extraction method, differences in environmental conditions over the years may have affected the structure and composition of sericin, thereby influencing its bioactivity [3].

The antiproliferative results observed in this study are consistent with the findings of Kaewkorn et al. [34], who demonstrated that sericin, at a concentration of 1.6 mg/mL, reduced the proliferation of the human colorectal cancer cell line (SW480). Similarly, although at a higher concentration, Kumar and Mandal [35] reported a reduction in cell growth with 4 mg/mL of sericin in various cell lines, including keratinocyte (HaCaT), non-tumorigenic epithelial (MCF-10), human squamous cell carcinoma (A431), and human tongue carcinoma (SAS) cell lines. However, no significant effect was observed at 1 mg/mL. On the other hand, Hakimi et al. [36] evaluated sericin from different species (*B. mori* and *Antheraea pernyi*) and found that sericin from *A. pernyi* exhibited stronger cytotoxic effects, which were mitigated by enzymatic treatment. This suggests that the presence of toxic peptides in cocoons could be a defense mechanism against predators or microorganisms. These toxic peptides and serum components in the culture medium may possess sericin’s antiproliferative effects, as previously noted by other studies [37,38]. Therefore, the antiproliferative activity observed in the present study may be attributed to the presence of these toxic peptides in the cocoons, as well as the specific amino acid profile of sericin [39].

The antiproliferative activity observed in our study may similarly be linked to the presence of such peptides or, alternatively, to specific amino acids in sericin that exhibit cytotoxic properties. Amino acids such as lysine (Lys), arginine (Arg), and histidine (His) are known to induce cancer cell cytotoxicity under acidic conditions by increasing membrane permeability [40,41]. Additionally, Glu and Asp have been reported to exert antiproliferative effects on tumor cells [42]. Other amino acids, including Cys, proline (Pro), glicine (Gly), and phenylalanine (Phe), could interact with the target cell membranes, potentially enhancing the cytotoxic activity [43,44,45,46]. Tyr has also been shown to increase cytotoxicity [43], while tryptophan (Trp) can enter cancer cells through endocytic pathways [47,48].

In this study, several of these amino acids were identified in both extracted and commercial sericin samples. Notably, Glu, Gly, His, Tyr, and Trp were present in commercial sericin samples, with Lys additionally found in SC2. The extracted samples contained common amino acids such as Glu, Gly, and Trp, with S1 also exhibiting Tyr and Phe, while S2 and S3 contained His and Tyr. Sample S4 stood out with the presence of Lys, Asp, and Cys. Identifying these amino acids, particularly Cys and Asp, in sample S4 could explain its superior antiproliferative activity compared to other samples. In contrast, the lower number of identified amino acids in commercial sericin (SC1-2), possibly due to differences in its extraction methods, may have contributed to its reduced interaction with tumor cells and lower antiproliferative potential.

Overall, the distinct amino acid profiles of the sericin samples suggest that specific amino acids may play a crucial role in mediating their antiproliferative effects. Notably, sample S4 demonstrated the most promising results, likely due to its high concentration of Cys, which was uniquely abundant in this sample. In contrast, the other extracted samples, and especially the commercial samples, exhibited higher levels of Tyr but showed no significant antiproliferative potential. The commercial sericin samples contained the highest Tyr concentrations, ranging between 85% and 78%, and lacked any measurable antiproliferative effects. By comparison, the extracted samples, excluding S4, had Tyr levels ranging from 53% to 63%, further supporting the hypothesis that the presence of Cys in S4 may be a key contributor to its superior antiproliferative activity.

To the best of our knowledge, no previous reports evaluate the antioxidant activity of sericin using the thiobarbituric acid reactive substances (TBARS) method. However, other studies have demonstrated the antioxidant potential of sericin through different assays. For example, Manosroi et al. [49] reported on the free radical scavenging activities of five Thai native silk varieties using the DPPH assay, with SC50 values ranging from 13.65 ± 0.20 mg/mL to 54.49 ± 0.59 mg/mL. Saha et al. [12] also demonstrated the antioxidant activity of *B. mori* silk cocoons at 20 mg/mL and 40 mg/mL concentrations. Similarly, Kumar and Mandal [50] showed the antioxidant capacity of sericin from *Antheraea assamensis*, *Philosamia ricini*, and *B. mori* using FRAP and DPPH assays, as well as in vitro methods such as the Lactate Dehydrogenase (LDH) assay to determine the intracellular antioxidant activity. Further supporting these findings, Manesa et al. [13] demonstrated the antioxidant activity of sericin from Southern African cocoons using DPPH, FRAP, and ABTS assays. These studies highlighted the influence of the biochemical profile, cocoon origin, and extraction method on sericin’s antioxidant properties, which parallels the findings in the present study. They also recommended using complementary assays to more accurately assess the antioxidant potential, particularly in relation to lipid oxidation inhibition and free radical scavenging capacity.

In the current study, the antioxidant activity of sericin was also evaluated using the Cellular Antioxidant Activity (CAA) assay in RAW 246.7 cells (macrophage cell line, established from a tumor in a male mouse induced with the Abelson murine leukemia virus) at a concentration of 2 mg/mL, as higher concentrations were found to be toxic and were, therefore, excluded from further testing. The results indicated that the sericin samples did not exhibit antioxidant activity. Few studies have evaluated the antioxidant potential of sericin in cellular models, but some investigations have shown its effect on intracellular reactive oxygen species (ROS) generation. For instance, Kumar et al. [51] studied sericin’s effect on ROS production in human keratinocytes (HaCaT), and Kumar and Mandal [21] examined mouse melanoma (B16F10) cells. In these studies, cells were incubated with sericin or vitamin C before UVA (8 J/cm^2^) and UVB (120 mJ/cm^2^) radiation exposure. Sericin from non-mulberry silk varieties (*A. assamensis* and *P. ricini*) significantly reduced ROS production at 10 μg/mL in HaCaT cells, and in B16F10 cells, sericin from *A. assamensis* reduced ROS levels under both UVA and UVB conditions, while sericin from *P. ricini* was effective only under UVB exposure.

The antioxidant activity of sericin has been linked to its amino acid composition. According to Fatahian et al. [52] and Lamboni et al. [53], the presence of serine and threonine, whose hydroxyl groups chelate trace elements like copper and iron, contributes to this activity. However, in the non-hydrolyzed samples, serine and threonine were not identified in large quantities, which may explain the absence of antioxidant activity observed in this study.

Similarly, sericin did not exhibit anti-inflammatory activity, which supports findings from other reports. For instance, Panilaitis et al. [54] examined the response of the RAW 264.7 cell line stimulated with LPS to silk protein extracted using 0.02 M Na_2_CO_3_. They concluded that sericin in its soluble form lacked inflammatory activity, while sericin bound to fibers could induce inflammatory reactions. Kundu et al. [55] suggested that sericin-coated fibers might promote macrophage adhesion or prepare them for subsequent stimulation, possibly due to conformational changes occurring when sericin binds to silk fibers.

The antimicrobial activity was determined using several bacteria via a colorimetric test. Our results align with previous studies that have reported sericin’s antimicrobial activity against Gram-positive and Gram-negative bacteria from various sources. Manesa et al. [13] observed antimicrobial effects against Gram-positive bacteria for sericin extracted from *Gonometa postica*, *Gonometa rufobrunnae*, and *Argema mimosa* cocoons at concentrations between 5 and 10 mg/mL using the agar well diffusion assay. Their findings showed activity against three Gram-positive bacteria (*Bacillus subtilis*, *Staphylococcus aureus*, and *Staphylococcus epidermidis*) and two Gram-negative strains (*Escherichia coli* and *Salmonella enterica*). Similarly, Baptista-Silva et al. [56] evaluated the antimicrobial potential of a sericin solution and a sericin-based hydrogel derived from cocoons in Castelo Branco, Portugal, using a well diffusion assay against *S. aureus*, *P. aeruginosa*, and *E. coli*. This study found that neither the sericin solution nor the hydrogel inhibited bacterial growth, consistent with our findings for sample S1, which shares the same origin.

In contrast, Noosak et al. [57] demonstrated the antimicrobial efficacy of Thai silk sericin extracted via a chemical-free boiling method, with concentrations ranging from 40 to 320 μg/mL. The MIC and MBC were assessed against *S. aureus* ATCC29213 and clinical isolates (DS18, DS27, DS84, DS87, DS88, DS92, and DS110). Their results showed MIC and MBC values between 160 and 320 μg/mL when prepared using sterile saline, whereas inoculums prepared in broth exhibited MIC and MBC values above 320 μg/mL. This study suggests that the cocoon’s origin and the extraction method may influence the antimicrobial properties of sericin.

In addition, the antimicrobial activity of sericin may be associated with the presence of Cys, as it contains sulfhydryl groups as constituents [58], being identified only in sample S4. Sulfhydryl groups can form weak hydrogen bonds with oxygen or nitrogen, resulting in highly reactive compounds that impact the enzymatic reactions and metabolic functions of microorganisms [59]. However, our results and the studies analyzed could demonstrate that the cocoons’ harvesting time and the sericin extraction method could influence the samples’ antimicrobial potential. These variables should be carefully controlled in future studies to optimize the antimicrobial properties of sericin.

The anticoagulant potential of sericin was evaluated via PT and APTT assays. Sericin sample S4 and the two commercial samples (SC1 and SC2) showed more pronounced anticoagulant activity than the other sericin samples. Our findings indicate that sericin can potentially prolong the coagulation time, a result that diverges from the study by Sano et al. [60], which reported that only sulfated sericin exhibited anticoagulant activity greater than heparin, a well-known sulfated polysaccharide. The mechanism behind this difference could be attributed to the hydroxyl groups in serine, which play a pivotal role in binding large quantities of sulfate during sericin sulfation. Tamada et al. [61] also demonstrated that sulfated sericin has anticoagulant properties, but its efficacy is strongly influenced by its molecular weight, as Monti et al. [62] highlighted. This suggests that sericin’s sulfation level and molecular size can impact its anticoagulant potential, pointing to a complex interplay of factors that warrant further investigation.

Regarding anticoagulant mechanisms, sericin may be acting at multiple points within the coagulation cascade. The inhibition of thrombin or Factor Xa—both critical enzymes in the formation of fibrin clots—could be involved, as could interference with vitamin K-dependent factors by altering their synthesis or calcium-binding properties [63]. However, pinpointing the exact pathway of sericin’s anticoagulant effect remains a subject for future research.

Interestingly, although sericin extended the clotting times across both assays, significant differences emerged in the APTT assay compared to the control, suggesting that sericin primarily exerts its anticoagulant effect through the intrinsic pathway. This is consistent with its interaction with factors involved in the internal clotting mechanism, although the exact targets remain to be identified. In this regard, our results position sericin as a potential candidate, although further studies are essential to fully elucidate its mechanism of action as an anticoagulant agent.

On the other hand, the biocompatibility of sericin was confirmed using the MTT assay with human fibroblasts at concentrations below 1.66 mg/mL. These findings suggest that sericin is potentially biocompatible depending on the concentration used. However, several researchers highlight that the extraction method plays a key role in determining sericin’s biocompatibility. According to the literature, different extraction techniques yield sericin with varying molecular weights, which influence its chemical and biological properties [26]. For instance, Aramwit et al. [64] reported that the physical extraction method produces sericin with molecular weights between 25 and 150 kDa, whereas chemical methods yield molecular weights of 50–150 kDa (acid degradation) and 15–75 kDa (alkaline degradation). Moreover, urea-based extraction is not recommended for biological applications [26]. These variations in molecular weight are directly linked to sericin’s biological effects, as media containing lower-molecular-weight sericin have been shown to promote higher cell viability (>92%) [65].

The molecular weight of sericin also appears to correlate with the proportion of α and β subunits within its structure. The β subunit is characterized by lower hydrogen and carbon contents and higher nitrogen and oxygen levels [22]. Higher-molecular-weight sericin contains a greater amount of β subunits, which hinders the solubility of sericin and, consequently, its absorption by cells [22,66].

These considerations align with the findings of this study. Although the molecular weight was not explicitly measured, the sericin used in this work likely had a high molecular weight, as it was extracted via the physical method. This may explain the lower cell viability (<70%) observed at higher concentrations, likely due to limited solubility, which compromises cellular interaction.

It was also observed that sericin, at lower concentrations (<1.66 mg/mL), does not exhibit cytotoxic effects, in agreement with the findings of Aramwit et al. [26]. Interestingly, at a concentration of 0.33 mg/mL, the cell viability surpassed 100% compared to the negative control (cells cultured in medium with 10% FBS). Similarly, Zhang et al. [67] reported that a 15 μg/mL sericin concentration produced better results than the control group. Thus, the concentration of sericin significantly influences its biological activity, with lower concentrations enhancing cell proliferation, although the precise mechanism behind this remains unclear.

Finally, the ability of sericin to induce fibroblast migration was evaluated in this study. The analysis of the results shows that sericin induces fibroblast migration at all tested concentrations, with no significant differences observed between them. These findings are consistent with those reported by Martinez-Mora et al. [68], who demonstrated that sericin significantly promotes cell migration into the created gap when compared to cells incubated with culture medium supplemented with 10% FBS. Similarly, this effect has been corroborated by other studies in the literature. For instance, Zhang et al. [67] found that sericin concentrations of 15 μg/mL showed better results compared to the control group (cells exposed to 10% FBS). In the same way, Santos [69] observed that 13.95 × 10^−1^ ng/μL of sericin (equivalent to 0.10%) stimulated cell migration, resulting in a notable reduction of the “wound” area in the scratch assay.

The ability of sericin to induce cell migration has also been investigated by Martínez-Mora et al. [68], who explored the molecular mechanisms responsible for sericin’s effects on wound healing. The authors performed a scratch assay using various concentrations of sericin.

Based on the results, sericin plays a significant role in wound healing by promoting fibroblast migration to the wound site. Once present at the injury site, these cells begin secreting extracellular matrix proteins, particularly collagen, that are crucial for forming new tissue. Furthermore, fibroblasts are involved in the secretion of various cytokines that stimulate and regulate the activity of other cells (e.g., immune cells, keratinocytes, endothelial cells, etc.). However, the exact mechanism through which sericin stimulates cell migration remains unclear in the literature. According to Martínez-Mora et al. [68], sericin induces the production of PAI-1, a gene involved in cell migration. Nevertheless, further research is needed to better understand the biological and physicochemical properties of sericin, as they hold significant potential in biomedicine, particularly regenerative medicine. In addition, these promising results can be an avenue to explore the use of sericin as an alternative supplement to the FBS for cell culture.

## 4. Materials and Methods

### 4.1. Material

Reagents and solvents were purchased from a commercial source to realize this study. FBS, glutamine, penicillin, streptomycin, RPMI-1640 medium, and Hanks’ balanced salt solution (HBSS) were obtained from Hyclone (Logan, UT, USA). DMEM medium was obtained from Gibco Invitrogen Life Technologies (Carlsbad, CA, USA). 2.2 2′-azobis(2-methylpropionamide) dihydrochloride (AAPH) solution (600 μM) was purchased from Panreac Applichem (Barcelona, Spain). Acetic acid, FeSO4, ellipticine, sulforhodamine B (SRB), the nitrite calibration curve, trypan blue, trichloroacetic acid (TCA), and Tris(hydroxymethyl)aminomethane (Tris) were purchased from Sigma Chemical Co. (St. Louis, MO, USA). Tris-(hidroximetil)-aminometano)-HCl (Tris-HCl), thiobarbituric acid (TBA), quercetin, liposaccharide solution (LPS), dexamethasone, dimethyl sulfoxide (DMSO), p-iodonitrotetrazolium chloride (INT), ascorbic acid, methicillin, and ampicillin were acquired from Sigma-Aldrich (St Louis, MO, USA). 2′,7′-dichlorohydrofluorescein (DCFH) was obtained from Acros Organics (Geel, Belgium). The Griess Reagent System kit (nitrophenamide, ethylenediamine, and nitrite solutions) was from Promega, Madison, WI, USA. The fungicide ketoconazole, Tween 80, and Malt Extract Broth (MEB) were from Frilabo, Porto, Portugal. The Tryptic Soy Broth (TSB) was from Biomerieux (Marcy l’Etoile, France). The blood agar (7% sheep blood) was obtained from Liofilchem (Roseto Degli Abruzzi, Italy). APTT and CaCl2 were obtained from HORIBA ABX SAS, Portugal Branch (Amadora, Portugal). Ortho-phthalaldehyde (OPA), fluorenylmethyloxycarbonyl chloride (FMOC-Cl), 3-mercaptopropionic acid derivatizer (3-MPA), acetate buffer solution, and sodium acetate were acquired from Sigma-Aldrich (Darmstad, Germany). The amino acid standards Asp, Arg, Asn, Cys, Hydroxyproline (Hyp), His, Ile, Leu, Lys, Methionine (Met), Pro, Ser, Tau, threonine (Thr), and Val were purchased from Sigma-Aldrich. The amino acid standards Glu, Ala, Phe, and Gly were purchased from Merck (Rahway, NJ, USA). And the amino acid standards Gln and Trp were acquired from Fluka (Everett, WA, USA). Triethylamine (TEA) was obtained from Carlo Erba (Val de Reuil, France). Acetic acid was purchased from Panreac (Barcelona, Spain). Pooled human plasma (blood-derived) Na citrate was obtained from Innovative Research (Novi, MI, USA).

Sericin standards were purchased from Sigma-Aldrich (St Louis, MO, USA) and FUJIFILM Wako Chemicals (Neuss, Germany) and were coded as CS1 and CS2, respectively.

### 4.2. Extraction of Sericin

The sericin was extracted from cocoons collected in different parts of Portugal. The Casa da Seda, integrated into the Centro de Ciência Viva de Bragança and the Museu da Seda de Freixo de Espada à Cinta, contributed to the cocoons that originated in Bragança district. Cocoons from 3 different years—2019, 2021, and 2022—were then donated, having received the codes S2, S3, and S4, respectively. The Portuguese Association of Parents and Friends of Mentally Handicapped Citizens (APPACDM) contributed cocoons from Castelo Branco for 2019 (S1).

The extraction process was described by da Silva et al. [66] and optimized. First, the cocoons were manually cleaned and divided into fragments measuring around 1 cm^2^. They were then rinsed three times with deionized water and tap water. For the degumming process, the cocoons were dried at 50 °C, weighed, and submerged in ultrapure water at a ratio of 1.5% w/V. An autoclave was used to extract the aqueous sericin solution at 120 °C for 30 min. The sericin solution was then filtered to remove the fibers, stored in a sealed container, and frozen at −80 °C. By lyophilizing the sericin solutions, sericin powders were obtained and kept at 4 °C until usage [70].

### 4.3. Chemical Characterization

Amino acid analysis was performed using high-performance liquid chromatography (HPLC) on a Shimadzu system (Tokyo, Japan), which included an SCL-40 system controller, LC-40D pump, CTO-40C column oven, four-channel low-pressure gradient unit, SIL-40C automatic injector, DGU-405 5-channel degasser, and RF-20Axs fluorescence detector (FLD). All the samples were analyzed with or without hydrolysis, as described in [71]. Two mL of 4 M methanesulfonic acid (MSA) with 0.2% tryptamine (to preserve the tryptophan concentration during acid hydrolysis) was added to 16 mm × 25 mm screw-cap tubes containing 20 mg of sample. The tubes were sealed with nitrogen, heated to 110 °C for a whole day in an electric oven, and allowed to cool, and then their contents were vacuum filtered through Whatman No. 4 paper. The filtrate was diluted to 4 mL in a glass vial with ultrapure water, and 1 mL of the resulting liquid was membrane-filtered (using Millipore^®^ 0.45 μm regenerated cellulose membranes, Merck, Darmstadt, Germany). Reverse-phase high-performance liquid chromatography (RP-HPLC) with fluorescence detection was then used to determine the total amino acid content. Two hydrolyses of each sample were performed.

The HPLC-FLD analysis of total and free amino acids involves a derivatization step using OPA and FMOC-CI, as described in [71] and adapted to a micro-well plate [71]. The derivatization process was carried out in a 1.5 mL amber vial and involved adding 80 µL of the sample or standard to 200 µL of the internal standard solution (norvaline), 200 µL of borate buffer (80 mM boric acid, pH = 10.4, VWR), 80 µL of the derivatized OPA/3-MPA, and 8 µL of the derivatized FMOC-Cl dissolved in acetonitrile.

The derivatized compounds were separated using a Kinetex Core-Shell C18 column (5 µm, 150 mm × 4.6 mm, Phenomenex, Torrance, CA, USA), with a 10 µL sample injected. The mobile phase was prepared from a gradient of two solutions: A (25 mM acetate buffer solution—2.05 g sodium acetate in 1 L of water—and 0.05% TEA adjusted to pH 7.2 with concentrated acetic acid) and B (mixture of water/acetonitrile (Chromasolv, VWR)/methanol (HPLC gradient, VWR) (20:40:40, *v*/*v*)).

Amino acid detection was carried out via fluorescence and was measured for amino acid derivatives, OPA-3MPA (λex = 340 nm, λem = 450 nm), and FMOC-Cl derivatives (λex = 237 nm, λem = 340 nm, greater sensitivity for proline), with a change in λ at 26.25 min. The data were presented in mg/L, with norvaline serving as the standard. All samples were digested, extracted in duplicate, and injected in duplicate.

### 4.4. Bioactivity Analysis

#### 4.4.1. Cell Lines and Culture Conditions for Cellular Assays

To realize the different in vitro bioactivities, we used tumor cell lines and non-tumor cell lines—AGS, CaCo-2, NHDF, and RAW 264.7—which were purchased from the European Collection of Authenticated Cell Cultures (ECACC), and MCF-7, HeLa, and NCI-H460, which were acquired from the Leibniz-Institute DSMZ–German Collection of Microorganisms and Cell Cultures GmbH (Braunschweig, Germany). All the cell lines were maintained in RPMI-1640 medium supplemented with 10% FBS, glutamine (2 mM), penicillin (100 U/mL), and streptomycin (100 mg/mL), individually, with all being obtained from Hyclone (Logan, UT, USA), with the exception of Vero, NHDF, and RAW 246.7, which were maintained in DMEM medium, obtained from Gibco Invitrogen Life Technologies (Carlsbad, CA, USA), supplemented with fetal bovine serum (10%), glutamine, and antibiotics. Only when the cells had achieved 70 to 80% confluence could they be utilized; until then, the culture flasks were incubated in an incubator under a humid atmosphere, at 37 °C and with 5% CO_2_ (Heal Force CO_2_ Incubator, Shanghai Lishen Scientific Equipment Co, Ltd., Shanghai, China).

#### 4.4.2. Antiproliferative Activity

The antiproliferative activity was analyzed using the SRB assay, according to [72], in five human tumor cell lines: AGS, CaCo-2, MCF-7, HeLa, and NCI-H460. Using a stock solution of the sericin samples with a 16 mg/mL concentration, four concentrations between 1.6 and 0.025 mg/mL were achieved through a series of dilutions. The percentage of extract concentration that inhibits cell growth in 50% (GI_50_ in µg/mL) represents the way the results are discussed. To obtain the results, the microplate reader Synergy H1 (BioTek Instruments, Winooski, VT, USA) was used to measure the absorbance at a wavelength of 540 nm. Ellipticine was used as a positive control.

#### 4.4.3. Antioxidant Activity

##### Thiobarbituric Acid Reactive Substances (TBARS) Method

The antioxidant potential of sericin was determined through the inhibition of lipid peroxidation using TBARS, described in [73]. Sericin samples weighing 140 mg each were diluted in 2 mL of distilled water to create stock solutions with a 70 mg/mL concentration. After a series of dilutions, six concentrations ranging from 2.18 mg/mL to 70 mg/mL were obtained from stock solutions. The peroxidation inhibition percent was obtained by using the following formula:

% lipid peroxidation inhibition = (A − B)/A × 100%(1)

where A and B were the absorbance of the control and the compound solution, respectively. The absorbance was measured at 532 nm. Tris-HCl buffer and distilled water were used as a control.

##### Cell Antioxidant Activity (CAA) Assay

CAA, as described in [74], was used to evaluate the antioxidant potential of sericin in a cellular model. In this procedure, RAW 246.7 was used, a mouse macrophage cell line. The sericin samples were dissolved in H_2_O to a concentration of 8 mg/mL, and then successive dilutions were made with DCFH to obtain the concentrations to be tested (500–2000 μM). Fluorescence was measured using a Biotek Synergy H1 microplate reader at 470 nm excitation and 530 nm emission every five minutes for an hour. DCFH and DMEM culture medium were used as a negative control, while quercetin was utilized as a positive control.

#### 4.4.4. Anti-Inflammatory Activity

Nitric oxide production (NO) inhibition, as described in [75], was used to assess the anti-inflammatory potential of sericin. The RAW 264.7 cell line was used in this analysis. Each sericin sample was weighed and diluted in 1 mL of sterile water to produce stock solutions with a 16 mg/mL concentration. After a series of dilutions, four concentrations between 1.6 and 0.02 mg/mL were obtained from the stock solutions. A microplate reader (BioTek Synergy H1) was used to assess the absorbances at 540 nm, and the amount of nitric oxide produced was determined by comparing the values obtained to the calibration line for reference. Dexamethasone was used as a positive control, and samples without LPS were used as a negative control.

#### 4.4.5. Antimicrobial Activity

##### Preparation of Bacterial Inoculum

Clinical bacteria were isolated at the Hospital Center of Trás-os-Montes and Alto Douro (Vila Real, Portugal) from patients being treated in several departments. The five Gram-negative bacteria tested in this analysis were *Escherichia coli*, *Proteus mirabilis*, and *Klebsiella pneumoniae*, isolated in urine, as well as *Pseudomonas aeruginosa* and *Morganella morganii*, isolated in sputum; three Gram-positive bacteria—*Enterococcus faecalis* (urine), *Listeria monocytogenes* (isolated from cerebrospinal cord fluid), and methicillin-resistant *Staphylococcus aureus* (MRSA)—isolated in sputum, were used. Two positive controls were produced: one with medium, bacteria, and antibiotics and the other with TSB and each inoculum.

##### Antibacterial Activity

To prepare a stock solution with a final concentration of 20 mg/mL, the samples were initially dissolved in a solution containing 5% (*v*/*v*) DMSO and 95% autoclaved distilled water. Subsequently, the concentration ranges were achieved through a serial dilution of the samples, ranging from 10 to 0.03125 mg/mL. A colorimetric test was used to determine the MIC on all bacteria, as described in [76]. The MIC was defined as the lowest quantity that inhibited the observable bacterial growth, as demonstrated by a change in color from yellow to pink if the microorganisms were viable. The smallest concentration at which no growth occurred was known as the MBC. The minimal concentration required to annihilate microorganisms is the MBC. Streptomycin and ampicillin were utilized as a positive control.

#### 4.4.6. Anticoagulant Activity

The procedure was followed according to the guidelines in the manual of the Yumizen G200 coagulation analyzer (HORIBA ABX SAS, Montpellier, France). The anticoagulant activity was evaluated using the APTT and PT methods. For testing, the highest concentration (1.6 mg/mL) was achieved by dissolving a known quantity of each extract (0.8 mg) in 0.5 mL of pooled human plasma (blood-derived) Na citrate and centrifuged at 500× *g* for 5 min at 4 °C. Subsequently, dilutions were prepared to obtain a concentration of 0.025 mg/mL for assessment. The APTT assay was evaluated using 50 µL of sample and 50 µL of APTT mixed in a test tube and incubated for 3 min at 37 °C. Then, 100 µL of CaCl2, was added to the mixture. The anticoagulant potential was determined based on the time required for clot formation. For the PT test, a 50 µL sample was added to a cuvette and incubated for 2 min at 37 °C in equipment, the PT solution was added, and the anticoagulant potential was determined based on the time required for clot formation.

#### 4.4.7. Cytotoxic Activity

The cytotoxicity of sericin was analyzed using the MTT method. The MTT method was carried out using sample S1 at the following concentrations: 6.65 mg/mL, 5.32 mg/mL, 3.33 mg/mL, 1.66 mg/mL, 1 mg/mL, and 0.33 mg/mL at 24, 48, and 72 h, following regulatory compliance with ISO 10993-5 [77].

NHDF cells were seeded in 96-well plates and incubated with sericin solutions for 24, 48, and 72 h. The MTT test was conducted to promote the reaction, with cells incubated with MTT (5 mg/mL) for 4 h at 37 °C. Formazan crystals were then dissolved in DMSO solution for 30 min under agitation. The absorbance of each well was determined at 570 nm using a spectrophotometer. The positive control had the lowest absorbance value, as cells were dead in DMEM-F12 medium supplemented with 10% FBS, while the negative control had the highest absorbance value, as cells were incubated only with DMEM-F12 culture medium supplemented with 10% FBS. Optical microscopy images were acquired to assess the morphology and proliferation of the cells before incubating the MTT solution with the cells.

#### 4.4.8. Evaluation of Sericin’s Ability to Promote Cell Migration (Scratch Assay)

The ability of sericin to induce the migration of human fibroblasts was evaluated using the scratch assay, adapted from [78,79]. Human fibroblasts were seeded in 12-well plates at a density of 80,000 cells/well and incubated at 37 °C, 5% CO_2_. A space was created using a sterile pipette tip, simulating a wound. Different concentrations of sericin (3.33 mg/mL, 1.66 mg/mL, and 1 mg/mL) were diluted in DMEM medium without FBS to evaluate the individual effect of sericin on the migratory activity of fibroblasts. A control group was also evaluated, which was incubated with DMEM-F12 medium supplemented with 10% FBS. Optical microscopy images were acquired at different incubation times, and the width of the created area was quantified using ImageJ software 1.51n.

### 4.5. Statistical Analysis

The findings of each test performed in this study, which was conducted in duplicate, are shown as mean values ± standard deviation.

ANOVA was used to examine the findings, and then the Turkey test was performed with a significance level of 0.05. GraphPad 5.0 software and SPSS v. 23.0 software were used to conduct statistical analyses.

## 5. Conclusions

This study compared sericin extracted from Portuguese silk cocoons with commercial sericin products across several bioactivities. The results indicated that origin and seasonality affect both the extraction yield and amino acid composition and, consequently, bioactivities. Non-hydrolyzed samples displayed a broader amino acid range, with higher levels of certain amino acids such as tyrosine, cysteine, serine, and glutamic acid, which may enhance specific bioactivities, including antiproliferative effects. Notably, the sericin sample S4 demonstrated superior antiproliferative potential, likely due to its amino acid content, including cysteine, which is known for its cytotoxic properties. The antimicrobial properties varied, likely due to the extraction method and cocoon origin, revealing to be related to the cysteine content of the samples. Additionally, sericin exhibited anticoagulant activity, particularly through the intrinsic coagulation pathway, making it a promising candidate for anticoagulant applications. Moreover, sericin’s antioxidant and anti-inflammatory activities were minimal, with no significant antioxidant effect observed; however, sericin was biocompatible, promoting fibroblast migration, supporting future exploitation for wound-healing applications.

In summary, this study highlights sericin’s potential for selective bioactivities, influenced by the amino acid composition and extraction variables. Our findings suggest sericin’s usefulness in wound healing and anticoagulation, although its low antioxidant and anti-inflammatory effects may limit broader biomedical applications. Further research is warranted to optimize sericin’s bioactivities and clarify its molecular mechanisms, particularly for clinical and therapeutic applications. Future studies will focus on a single bioactivity and explore alternative extraction methods to enhance the specificity for each bioactivity, with additional in-depth assays.

## Figures and Tables

**Table 1:** Amino acid profiles of different sericin samples.

	Free Amino Acid Content (%)						Total Amino Acid Content (%)					
	**SC1**	**SC2**	**S1**	**S2**	**S3**	**S4**	**SC1**	**SC2**	**S1**	**S2**	**S3**	**S4**
Asp	n.a.	n.a.	n.a.	n.a.	n.a.	6.73	10.97	12	10.77	9.45	9.53	9.62
Cys	n.a.	n.a.	n.a.	n.a.	n.a.	79.16	n.a.	n.a.	n.a.	n.a.	n.a.	n.a.
Glu	5.79	3.91	45.4	43.02	9.76	19.35	8.84	8.86	9	7.6	7.9	8.04
Ser	56.35	39.44	34.47	34.46	53.18	n.a.	59.48	59.02	54.05	56.44	56.67	55.74
Gly	28.28	18.9	12.66	13.21	21.29	27.31	7.01	6.2	1.5	6.38	5.91	5.79
His	2.92	2.06	n.a	1.61	1.58	6.29	2.16	2.04	2.11	2.07	2.07	2.11
Thr	n.a.	n.a.	n.a.	n.a.	2.62	n.a.	2.73	2.7	3.13	2.54	2.31	2.44
Ala	n.a.	n.a.	1.29	0.78	1.21	n.a.	1.41	1.37	1.53	1.32	1.27	1.29
Lys	n.a.	31.08	n.a.	n.a.	n.a.	n.a.	1.98	1.99	1.98	1.88	2.05	2.01
Tyr	84.99	78.26	53.42	59.65	62.58	n.a.	n.a.	n.a.	8.13	7.07	6.85	7.22
Val	n.a.	n.a.	n.a.	0.82	1.58	n.a.	1.64	1.81	1.6	1.36	1.34	1.45
Trp	6.66	4.6	2.48	3.19	3.34	8.34	0.28	0.28	0.22	0.23	0.31	0.28
lle	n.a.	n.a.	n.a.	n.a.	2.32	n.a.	0.59	0.59	0.75	0.6	0.6	0.6
Leu	n.a.	n.a.	3.7	2.91	3.13	n.a.	0.54	0.52	0.75	0.62	0.73	0.71
Arg	n.a	n.a	n.a	n.a	n.a	n.a	2.37	2.43	2.79	2.32	2.36	2.43
Phe	n.a	n.a	n.a	n.a	n.a	n.a	n.a.	0.19	0.3	0.24	0.26	0.26

n.a.: not available.

**Table 2:** Antiproliferative activities of sericin extracts determined via SRB assay.

	S1	S2	S3	S4	SC1	SC2	Positive Control (µg/mL)
Antiproliferative activity (GI_50_, mg/mL)							
AGS	1.127 ± 0.089 ^a^	1.235 ± 0.023 ^a^	0.826 ± 0.014 ^b^	0.791 ± 0.024 ^b^	>1.6	>1.6	1.23 ± 0.02
CaCo-2	>1.6	>1.6	1.025 ± 0.093 ^a^	0.814 ± 0.030 ^b^	>1.6	>1.6	1.21 ± 0.02
MCF-7	1.519 ± 0.028 ^a^	>1.6	0.944 ± 0.058 ^b^	0.520 ± 0.032 ^c^	>1.6	>1.6	1.02 ± 0.02
NCI-H460	0.803 ± 0.005 ^a^	0.682 ± 0.146 ^a^	0.656 ± 0.073 ^a^	0.667 ± 0.043 ^a^	>1.6	>1.6	1.01 ± 0.01
HeLa	>1.6	>1.6	1.072 ± 0.011 ^a^	1.142 ± 0.098 ^a^	>1.6	>1.6	1.03 ± 0.09

Results are expressed as the mean ± standard deviation. According to Tukey’s test at a level of significance of *p* < 0.05, averages that are followed by the same letters on the same line are not statistically different.

**Table 3:** Antibacterial activities of sericin extracts and commercial sericin on clinical bacteria.

**Antibacterial Activity (mg/mL)**								
Sample	Gram-Negative Bacteria					Gram-Positive Bacteria		
	*Enterobacter cloacae*	*Escherichia coli*	*Pseudomonas* *aeruginosa*	*Salmonella* *enterica*	*Yersinia* *enterocolitica*	*Bacillus* *cereus*	*Listeria* *monocytogenes*	*Staphylococcus aureus*
	MIC/MBC	MIC/MBC	MIC/MBC	MIC/MBC	MIC/MBC	MIC/MBC	MIC/MBC	MIC/MBC
S1	>10/>10	10/>10	>10/>10	>10/>10	10/>10	>10/>10	>10/>10	>10/>10
S2	>10/>10	10/>10	>10/>10	>10/>10	10/>10	>10/>10	>10/>10	>10/>10
S3	>10/>10	5/>10	10/>10	10/>10	10/>10	>10/>10	10/>10	10/>10
S4	10/>10	5/>10	10/>10	10/>10	10/>10	>10/>10	10/>10	10/>10
SC1	>10/>10	10/>10	10/>10	>10/>10	>10/>10	>10/>10	>10/>10	10/>10
SC2	>10/>10	10/>10	>10/>10	>10/>10	>10/>10	>10/>10	>10/>10	>10/>10
Streptomycin	0.007/0.007	0.01/0.01	0.06/0.06	0.007/0.007	0.007/0.007	0.007/0.007	0.007/0.007	0.007/0.007
Ampicillin	0.15/0.15	0.15/0.15	0.63/0.63	0.15/0.15	0.15/0.15	n.t./n.t.	0.15/0.15	0.15/0.15

n.t.: not tested. Positive control concentrations: streptomycin (1 mg/mL), methicillin (1 mg/mL), and ampicillin (10 mg/mL).

**Table 4:** Anticoagulant activities of sericin extracts and commercial sericin determined via PT and APPT assays.

	PT (s)				APTT (s)			
	1.6 mg/mL	0.4 mg/mL	0.1 mg/mL	0.025 mg/mL	1.6 mg/mL	0.4 mg/mL	0.1 mg/mL	0.025 mg/mL
Control	21.1 ± 0.4 ^c^	21.1 ± 0.4 ^b^	21.1 ± 0.4 ^a^	21.1 ± 0.4 ^a^	34.3 ± 1.0 ^d^	34.3 ± 1.0 ^c^	34.3 ± 1.0 ^b^	34.3 ± 1.0 ^b^
S1	21.5 ± 0.6 ^a, b, c^	21.5 ± 0.1 ^a, b^	21.5 ± 0.5 ^a^	21.2 ± 0.3 ^a^	38.7 ± 0.8 ^a^	37.6 ± 0.5 ^a^	35.2 ± 1.0 ^b^	34.8 ± 0.2 ^b^
S2	21.9 ± 0.4 ^a, b^	21.7 ± 0.3 ^a^	21.5 ± 0.4 ^a^	21.0 ± 0.4 ^a^	35.8 ± 0.4 ^c^	35.8 ± 1.3 ^b^	35.3 ± 0.3 ^b^	35.0 ± 0.6 ^b^
S3	21.6 ± 0.3 ^a, b, c^	21.4 ± 0.5 ^a, b^	21.3 ± 0.5 ^a^	21.1 ± 0.4 ^a^	36.7 ± 1.0 ^b, c^	36.0 ± 0.4 ^b^	35.3 ± 0.4 ^b^	34.7 ± 1.3 ^b^
S4	22.0 ± 0.4 ^a^	21.8 ± 0.3 ^a^	21.4 ± 0.3 ^a^	20.3 ± 1.1 ^a^	38.7 ± 0.5 ^a^	37.4 ± 0.3 ^a^	37.2 ± 0.9 ^a^	36.8 ± 0.8 ^a^
SC1	21.4 ± 0.5 ^a, b, c^	21.3 ± 0.2 ^a, b^	21.1 ± 0.5 ^a^	20.9 ± 0.4 ^a^	38.1 ± 0.4 ^a, b^	37.8 ± 0.1 ^a^	37.2 ± 0.3 ^a^	36.5 ± 0.6 ^a^
SC2	21.2 ± 0.0 ^b, c^	21.0 ± 0.1 ^b^	21.0 ± 0.9 ^a^	20.7 ± 0.5 ^a^	38.2 ± 0.8 ^a^	37.3 ± 0.3 ^a^	37.0 ± 1.5 ^a^	36.7 ± 0.4 ^a^

Results are expressed as the mean ± standard deviation. According to Tukey’s test, at a level of significance of *p* < 0.05, averages followed by the same letters on the same line are not statistically different.

## Data Availability

Data are contained within the article.
